# Natural genetic variation in Arabidopsis for responsiveness to plant growth-promoting rhizobacteria

**DOI:** 10.1007/s11103-016-0442-2

**Published:** 2016-01-30

**Authors:** Paul C. A. Wintermans, Peter A. H. M. Bakker, Corné M. J. Pieterse

**Affiliations:** Plant–Microbe Interactions, Department of Biology, Faculty of Science, Utrecht University, Padualaan 8, 3584 CH Utrecht, The Netherlands

**Keywords:** *Arabidopsis thaliana*, Genome-wide association (GWA), Plant growth-promoting rhizobacteria (PGPR), *Pseudomonas simiae* WCS417r, Volatile organic compounds (VOCs)

## Abstract

The plant growth-promoting rhizobacterium (PGPR) *Pseudomonas simiae* WCS417r stimulates lateral root formation and increases shoot growth in *Arabidopsis thaliana* (Arabidopsis). These plant growth-stimulating effects are partly caused by volatile organic compounds (VOCs) produced by the bacterium. Here, we performed a genome-wide association (GWA) study on natural genetic variation in Arabidopsis for the ability to profit from rhizobacteria-mediated plant growth-promotion. To this end, 302 Arabidopsis accessions were tested for root architecture characteristics and shoot fresh weight in response to exposure to WCS417r. Although virtually all Arabidopsis accessions tested responded positively to WCS417r, there was a large variation between accessions in the increase in shoot fresh weight, the extra number of lateral roots formed, and the effect on primary root length. Correlation analyses revealed that the bacterially-mediated increase in shoot fresh weight is related to alterations in root architecture. GWA mapping for WCS417r-stimulated changes in root and shoot growth characteristics revealed 10 genetic loci highly associated with the responsiveness of Arabidopsis to the plant growth-promoting activity of WCS417r. Several of the underlying candidate genes have been implicated in important plant growth-related processes. These results demonstrate that plants possess natural genetic variation for the capacity to profit from the plant growth-promoting function of a beneficial rhizobacterium in their rhizosphere. This knowledge is a promising starting point for sustainable breeding strategies for future crops that are better able to maximize profitable functions from their root microbiome.

**Electronic supplementary material:**

The online version of this article (doi:10.1007/s11103-016-0442-2) contains supplementary material, which is available to authorized users.

## Introduction

Beneficial microbes in the microbiome of plant roots provide important services to the plant as they improve plant nutrition and provide protection against plant pathogens (Berendsen et al. 2012; Bulgarelli et al. 2013; Philippot et al. 2013). In recent years, microarray and next-generation sequencing technologies provided detailed insight into the composition and assembly of microbiota communities on and in plants roots (Bai et al. 2015; Bulgarelli et al. 2012; Hardoim et al. 2015; Lareen et al. 2016; Lebeis et al. 2015; Lundberg et al. 2012; Mendes et al. 2011). Genotypic variations in host plants can influence the composition of the root microbiome with important adaptive consequences for plant health (Haney et al. 2015; Pieterse et al. 2016). Evidence is accumulating that domestication of crop species changed root microbiome characteristics in a plant genotype-dependent manner (Bulgarelli et al. 2015; Peiffer et al. 2013). It has been postulated that during the process of plant domestication, plant breeding for high yields under conditions of high fertilizer input in soils has resulted in the erosion of plant genotypic traits involved in beneficial plant–microbe interactions (Pérez-Jaramillo et al. 2016). Hence, the identification of loci associated with the capacity of plants to maximize profitable functions from their root microbiome will be highly instrumental for sustainable plant breeding strategies that are directed towards optimizing root microbiome functions in crops.

Plant root-colonizing bacteria that have a positive effect on plant growth are collectively known as plant growth-promoting rhizobacteria (PGPR). Mechanisms of growth promotion have been studied in detail and different PGPR may have different modes of action. Many PGPR stimulate plant growth through the inhibition of plant pathogens. For some PGPR this is due to direct inhibition of growth and activity of pathogens (Doornbos et al. 2012; Raaijmakers and Weller 1998), whereas others can suppress diseases indirectly by eliciting induced systemic resistance (ISR), a process that is characterized by priming plant-borne defense mechanisms to respond faster and stronger to pathogen attack (Conrath et al. 2015; Pieterse et al. 2014). Numerous rhizosphere inhabiting bacteria can elicit ISR in plants, including *Pseudomonas* spp. (Bakker et al. 2007; Pieterse et al. 2014) and *Bacillus* ssp. (Kloepper et al. 2004). Also abiotic stresses like salinity and drought stress can be alleviated by PGPR (Yang et al. 2009). Finally, PGPR can exhibit a direct influence on plant growth and development (Lugtenberg and Kamilova 2009) and this phenomenon is the focus of this study.

A common way by which PGPR directly promote plant growth is by aiding in the uptake of essential nutrients. For example, *Rhizobium* species stimulate the formation of root nodules in which atmospheric nitrogen is fixed and made available to the plant (Van Rhijn and Vanderleyden 1995). Other PGPR free up scarce nutrients in the soil, like phosphate (Browne et al. 2009), or induce plant uptake mechanisms for nitrate (Mantelin and Touraine 2004) or iron (Zamioudis et al. 2014, 2015; Zhang et al. 2009). PGPR can also influence plant growth by the production of plant hormones like auxin, abscisic acid, cytokinins, and gibberellins (Hayat et al. 2010), or influence plant ethylene concentrations by production of the enzyme 1-aminocyclopropane-1-carboxylic acid (ACC) deaminase that breaks down ACC, the direct precursor of ethylene (Glick et al. 1998; Penrose et al. 2001). The latter process can effectively lower local ethylene concentrations resulting in increased root and shoot biomass (Glick et al. 2007).

More recently, volatile organic compounds (VOCs) have been reported to be involved in plant–microbe interactions, including rhizobacteria-mediated plant growth promotion (Blom et al. 2011; Junker and Tholl 2013). An early study reporting on VOCs and plant growth promotion deals with a *Bacillus subtilis* strain that promotes growth of *Arabidopsis thaliana* (Arabidopsis) via the bacterial VOCs acetoin and 2,3-butanediol (Ryu et al. 2003). Also root system architecture in Arabidopsis can be modulated by VOCs (Gutierrez-Luna et al. 2010). For instance, bacterial indole promotes root growth by interfering with auxin signaling in Arabidopsis (Bailly et al. 2014; Bhattacharyya et al. 2015). Moreover, Meldau et al. (2013) reported that the VOC dimethyl disulfide produced by *Bacillus* sp. B55 promotes growth in *Nicotiana attenuata*. Whereas most VOCs seem to act as inter-kingdom signaling molecules, dimethyl disulfide was suggested to be metabolized by the plant, thus reducing the need for energy costly sulfur uptake.

In Arabidopsis, colonization of the roots by the PGPR *Pseudomonas simiae* WCS417r (formerly known as *Pseudomonas fluorescens* WCS417r; Berendsen et al. 2015) can increase shoot fresh weight by approximately 30 % when co-cultivated in soil (Pieterse and Van Loon 1999). Using a split-plate system, it was shown that this growth-promoting effect is partly mediated by bacterial VOCs and unrelated to the ISR-inducing capacity of WCS417r (Zamioudis et al. 2013). Co-cultivating Arabidopsis accession Col-0 with WCS417r leads to inhibition of primary root elongation and promotion of lateral root and root hair formation, resulting in significant changes in the root architecture and increased shoot fresh weight (Zamioudis et al. 2013). This bacterially-induced process requires the action of the plant growth regulator auxin.

The involvement of microbial VOCs in plant growth promotion is typically studied by co-cultivating plants and PGPR in sealed Petri dishes in which microbially-produced CO_2_ can accumulate. Hence, it has been hypothesized that CO_2_ produced by the bacteria causes the growth promotion through the enhanced availability of this photosynthesis substrate. Whereas there is some evidence that CO_2_ can be partially involved (Kai and Piechulla 2009), the growth response stimulated by PGPR is far greater than can be explained by elevated CO_2_ alone (Blom et al. 2011). Elevated CO_2_ levels can increase plant biomass by up to 25 % (Sun et al. 2002; Ward and Strain 1997), but many PGPR easily surpass this as increases in plant biomass of over eightfold have been reported (Park et al. 2015). For WCS417r VOCs, Arabidopsis shoot fresh weight increases of up to fourfold have been reported (Zamioudis et al. 2013), indicating that also WCS417r VOCs have the capacity to stimulate plant growth beyond that caused solely by enhanced CO_2_ levels.

To investigate whether plant genotypic traits are involved in the plant’s capacity to profit from the plant growth-promoting activity of PGPR, we performed a genome-wide association (GWA) study. GWA mapping is a method initially utilized in human population studies to identify the genetic basis of complex traits (Hirschhorn and Daly 2005). GWA mapping has also been successfully utilized in plant studies (e.g. Aranzana et al. 2005; Atwell et al. 2010; Bac-Molenaar et al. 2015; Baxter et al. 2010; Chan et al. 2010; Kloth et al. 2012; Li et al. 2010). The underlying rationale of GWA studies is that natural variation in phenotypic traits in a population is caused by genetic differences that via single nucleotide polymorphisms (SNPs) in the genomes of the phenotyped genotypes can be linked to genetic loci and ideally candidate genes. Because plant growth promotion by PGPR is known to display host variation (Smith and Goodman 1999), GWA mapping may be a promising tool to investigate the genetic basis of PGPR-mediated plant growth promotion. In this study, we tested whether Arabidopsis has natural genetic variation in the ability to profit from the plant growth-promoting capacity of beneficial rhizosphere bacteria. If so, then this would hold promise for plant breeding strategies in crops that are aimed at maximizing profitable functions from the root microbiome. To test this, we used a global population of 302 Arabidopsis accessions that have been genotyped for 214 k SNPs (Baxter et al. 2010; Li et al. 2010) and performed a GWA study for growth-promotion characteristics in response to WCS417r. Virtually all accessions showed enhanced shoot fresh weight and increased lateral root formation upon exposure to WCS417r, while effects on primary root length were more variable. However, the magnitude of the plant growth-promoting effects was significantly different between Arabidopsis accessions and was independent of the intrinsic growth rate of the accessions. GWA mapping resulted in the identification of genetic loci associated with the plant’s capacity to benefit from growth-promoting effects of WCS417r rhizobacteria.

## Materials and methods

### Cultivation of bacteria

*Pseudomonas simiae* WCS417r (formerly known as *Pseudomonas fluorescens* WCS417r; Berendsen et al. 2015) was isolated in the 1980s from the rhizosphere of wheat (Lamers et al. 1988) and served since as a model *Pseudomonas* spp. strain for studying plant growth promotion (Van Peer and Schippers 1989; Zamioudis et al. 2013) and rhizobacteria-induced systemic resistance (Pieterse et al. 1996; Van Peer et al. 1991; Zamioudis and Pieterse 2012). WCS417r was grown for 24 h at 28 °C on King’s medium B agar medium supplemented with 50 µg mL^−1^ rifampicin as described (Van Wees et al. 2013). Subsequently, the WCS417r bacteria were suspended in 10 mL of 10 mM MgSO_4_ and centrifuged at 3200*g* for 5 min. The pellet was washed twice by resuspension in 10 mM MgSO_4_ and subsequent centrifuging at 3200*g* for 5 min after which the density was adjusted to 2 × 10^6^ colony-forming units (cfu) mL^−1^ of 10 mM MgSO_4_ (OD_600_ = 0.002).

### Plant growth conditions and PGPR treatment

A collection of 302 natural accessions of *Arabidopsis thaliana* (Supplemental Table S1) was used to investigate their responsiveness to growth-promoting effects of *P. simiae* WCS417r. Seeds were gas sterilized for 4 h in a desiccator as described (Van Wees et al. 2013). Sterilized seeds were sown on square Petri dishes with agar-solidified Murashige and Skoog (MS) medium supplemented with 0.5 % sucrose as described (Zamioudis et al. 2013). The seeds on MS agar were stratified for 2 days at 4 °C and subsequently incubated in a vertical position in a plant growth chamber at 21 °C with a 16-h day (100 µmol m^−2^ s^−1^) and 8-h night cycle. After 4 days of growth, seedlings of similar size were carefully transferred to 9 cm diameter Petri dishes containing MS agar medium. For the bacterial treatment, 240 µl of a WCS417r suspension containing 2 × 10^6^ cfu mL^−1^ was applied on the agar medium 5 cm below the roots of the seedlings. For the mock treatment, 240 µl of 10 mM MgSO_4_ was applied in a similar manner. The Petri dishes were briefly dried in a laminar flow cabinet, sealed with a lid and two layers of Parafilm, and placed in a vertical position in the growth room.

### Phenotyping responsiveness of Arabidopsis accessions to WCS417r

Per biological replicate, 4 seedlings of a tester Arabidopsis accession were grown on a MS agar plate alongside 4 seedlings of the reference accession Col-0. For each of the 302 tested accessions, 3 replicate plates with WCS417r-treated seedlings were compared to 3 replicate plates with mock-treated seedlings. After 8 days of growth, plates were photographed after which average primary root length per seedling and average number of lateral roots per seedling was determined for each plate. After 10 days of growth, average shoot fresh weight per seedling was determined for each plate of the mock- and WCS417r-treated seedlings. Data were obtained from three replicate plates per accession for both the control and the WCS417r treatment.

### GWA mapping, SNP selection, and statistics

A collection of 302 accessions was used to investigate the genetic variation present within Arabidopsis (Baxter et al. 2010; Li et al. 2010; Platt et al. 2010). Each of these accessions were genotyped versus the Col-0 accession with ~214 k single nucleotide polymorphism markers (Kim et al. 2007). GWA mapping was performed on the WCS417r-mediated changes in shoot fresh weight (∆SFW), number of lateral roots formed (∆LRF), and primary root length (∆PRL). For all traits, means per seedling (*n* = 4) per biological replicate (*n* = 3) were used to calculate the mean per treatment per accession. Values obtained after subtracting the mean value of the mock treatment from that of the WCS417r treatment were used for the GWA analysis (∆SFW, ∆LRF, ∆PRL). The online tool GWAPP (http://gwas.gmi.oeaw.ac.at/) was used for GWA mapping as described (Seren et al. 2012). The data sets were normally distributed and the AMM algorithm was employed for mapping. This algorithm closely resembles the commonly used EMMAX (Kang et al. 2010). The GWAPP Geneviewer was used to zoom in on trait-associated SNPs and reveal their position in the genome to pinpoint candidate genes within 10 kb up- and down-stream of the identified SNP. For each of the candidate genes, the annotations were retrieved from TAIR10 (arabidopsis.org). With the exception of the GWA mapping, all statistics were carried out with IBM SPSS Statistics 20.

## Results

### Natural variation in Arabidopsis for responsiveness to PGPR-mediated growth promotion

**Fig. 1:**
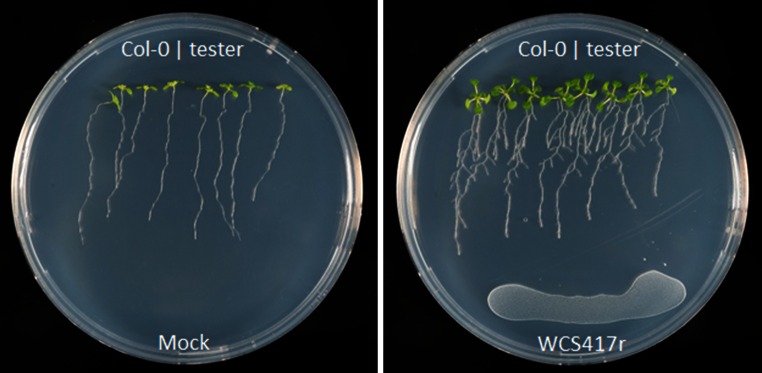
Phenotyping of Arabidopsis accessions for responsiveness to the plant growth-promoting effect of *P. simiae* WCS417r. Shown are photographs of MS agar plates with 4 Col-0 seedlings (*left half of the plate*) and 4 seedlings of a tester accession (*right half of the plate*), 8 days after spotting 240 µl of 10 mM MgSO_4_ (Mock) or a WCS417r bacterial suspension (2 × 10^6^ cfu mL^−1^) on the plate *below* the plant roots

**Fig. 2:**
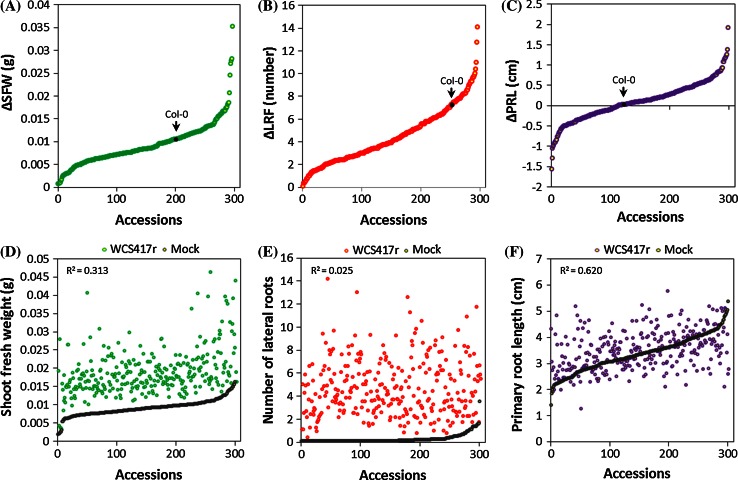
Natural variation of 302 *A. thaliana* accessions in their responsiveness to the growth-promoting effect of *P. simiae* WCS417r. **a** Accessions sorted for increase in shoot fresh weight (∆SFW) in response to exposure to WCS417r for 10 days (Col-0 is indicated with arrow and black dot). **b** Accessions sorted for increase in the number of lateral roots formed (∆LRF) in response to exposure of seedlings to WCS417r for 8 days. **c** Accessions sorted for difference in the length of the primary root (∆PRL) after exposure of the seedlings to WCS417r for 8 days. **d** Average shoot fresh weight of mock-treated (*grey dots*) and WCS417r-treated (*green dots*) plants. **e** Number of lateral roots formed in mock-treated and WCS417r-treated (*red dots*) plants. **f** Primary root length of mock- and WCS417r-treated (*purple dots*) plants. Each *dot* represents the average of 3 biological replicates. Values in **a**–**c** were calculated by subtracting the mean of the mock treatment from the mean of the WCS417r treatment. In each *panel*, accessions were sorted for increasing values. The order of the accessions differs per panel. The Pearson correlation coefficient (R^2^) indicates the degree of correlation between values of control and WCS417r-treated plants

In soil and plate assays, colonization of the roots of Arabidopsis accession Col-0 by *P. simiae* WCS417r has previously been shown to result in an increase in shoot fresh weight (Pieterse and Van Loon 1999; Zamioudis et al. 2013). On plates, it becomes visible that co-cultivation of Col-0 seedlings with WCS417r not only results in enhanced shoot growth, it also coincides with a faster development of lateral roots (Fig. 1), confirming previous findings (Zamioudis et al. 2013). In order to investigate the natural variation in responsiveness of Arabidopsis to the growth-promoting activity of WCS417r, we tested 302 Arabidopsis accessions for changes in shoot fresh weight and root architecture in response to exposure to WCS417r (Supplemental Table S1). PGPR-induced gain in shoot fresh weight (∆SFW), increase in the number of lateral roots formed (∆LRF), and effects on primary root length (∆PRL) were determined after an 8-day (root architecture) or 10-day (shoot fresh weight) period of co-cultivation with WCS417r by subtracting the average values of mock-treated plants from those of the WCS417r treatments. Figure 2a–c show that for all three parameters tested, the Arabidopsis accessions displayed extensive variation in the capacity to respond to WCS417r. Upon exposure to WCS417r, the reference accession Col-0 showed an average increase in shoot fresh weight of 10 mg, which is a 2.1-fold increase relative to the shoot fresh weight of mock-treated Col-0 plants. Among the 302 accessions, shoot fresh weight in response to WCS417r exposure changed between 0.9 and 4.2 fold, with Col-0 around the 65th percentile (Fig. 2a). For the increase in the number of lateral roots over the 8-day period of exposure to WCS417r, Col-0 performed in the upper range (Fig. 2b). While Col-0 formed on average 7 additional lateral roots in comparison to mock-treated seedlings, other accessions formed up to 14 additional lateral roots or none at all. The primary root length remained virtually unchanged in Col-0 in response to WCS417r, but in the other accessions it ranged from significant decreases to significant increases in length (Fig. 2c). Overall, these results demonstrate that Arabidopsis possesses extensive natural variation in responsiveness to the PGPR WCS417r.

### PGPR-mediated growth promotion capacity of Arabidopsis accessions is not linked to their intrinsic growth rates

To investigate whether the observed variation in the WCS417r-induced changes in the shoot and root growth parameters is related to intrinsic growth rates among the accessions, we plotted the absolute values of shoot fresh weight, number of lateral roots, and primary root length for both the mock and the WCS417r treatment and ranked the accessions ascendingly for the mock treatment (Fig. 2d–f). Figure 2d, e show that the values of the mock and the WCS417r treatment have a low correlation coefficient for the parameters shoot fresh weight and number of lateral roots (R^2^ = 0.313 and 0.025, respectively), indicating that the magnitude of these WCS417r-induced growth responses are, at the most, weakly related to the intrinsic growth capacity for the parameters tested under these conditions. Hence, faster growing accessions, or accessions that form more lateral roots in the experimental setup are not necessarily stronger responders to WCS417r. For the parameter primary root length, the correlation between mock and WCS417r treatment is stronger (R^2^ = 0.620; Fig. 2f).

### PGPR-mediated enhancement of shoot fresh weight is correlated with root architecture parameters

**Fig. 3:**
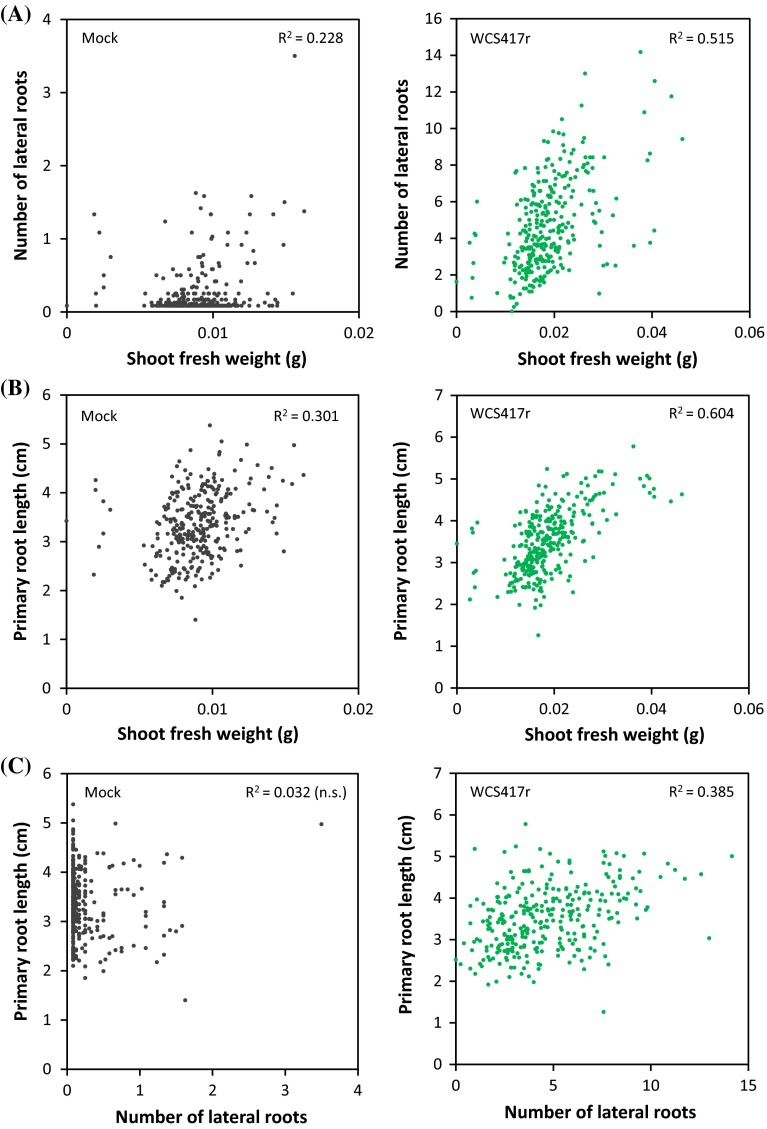
Correlation analysis of shoot fresh weight, number of lateral roots, and primary root length for 302 mock- and *P. simiae* WCS417r-treated Arabidopsis accessions. **a** Correlation between number of lateral roots and shoot fresh weight. **b** Correlation between primary root length and shoot fresh weight. **c** Correlation between primary root length and number of lateral roots. The correlation coefficient (R^2^) is presented in the *graphs* and is statistically significant at 0.01 (Pearson’s r, 2-tailed) unless stated otherwise (*ns* not significant)

In order to investigate whether the natural variation in the measured shoot and root growth characteristics of the 302 tested Arabidopsis accessions is correlated, we performed a correlation analysis for both the mock and the WCS417r data sets. Figure 3 shows that in mock-treated plants, shoot fresh weight only weakly correlates with the number of lateral roots (R^2^ = 0.228; Fig. 3a) and primary root length (R^2^ = 0.301; Fig. 3b), while primary root length and number of lateral roots do not correlate at all (R^2^ = 0.032; Fig. 3c). However, in WCS417r-treated plants, the correlation between number of lateral roots and shoot fresh weight (R^2^ = 0.515; Fig. 3a), primary root length and shoot fresh weight (R^2^ = 0.604; Fig. 3b), and primary root length and number of lateral roots (R^2^ = 0.385; Fig. 3c) is markedly higher than in the mock-treated plants. These results indicate that the PGPR-mediated increase in shoot fresh weight is at least partly related to PGPR-mediated changes in root architecture.

### GWA mapping reveals loci related to the capacity of Arabidopsis to respond to PGPR

**Fig. 4:**
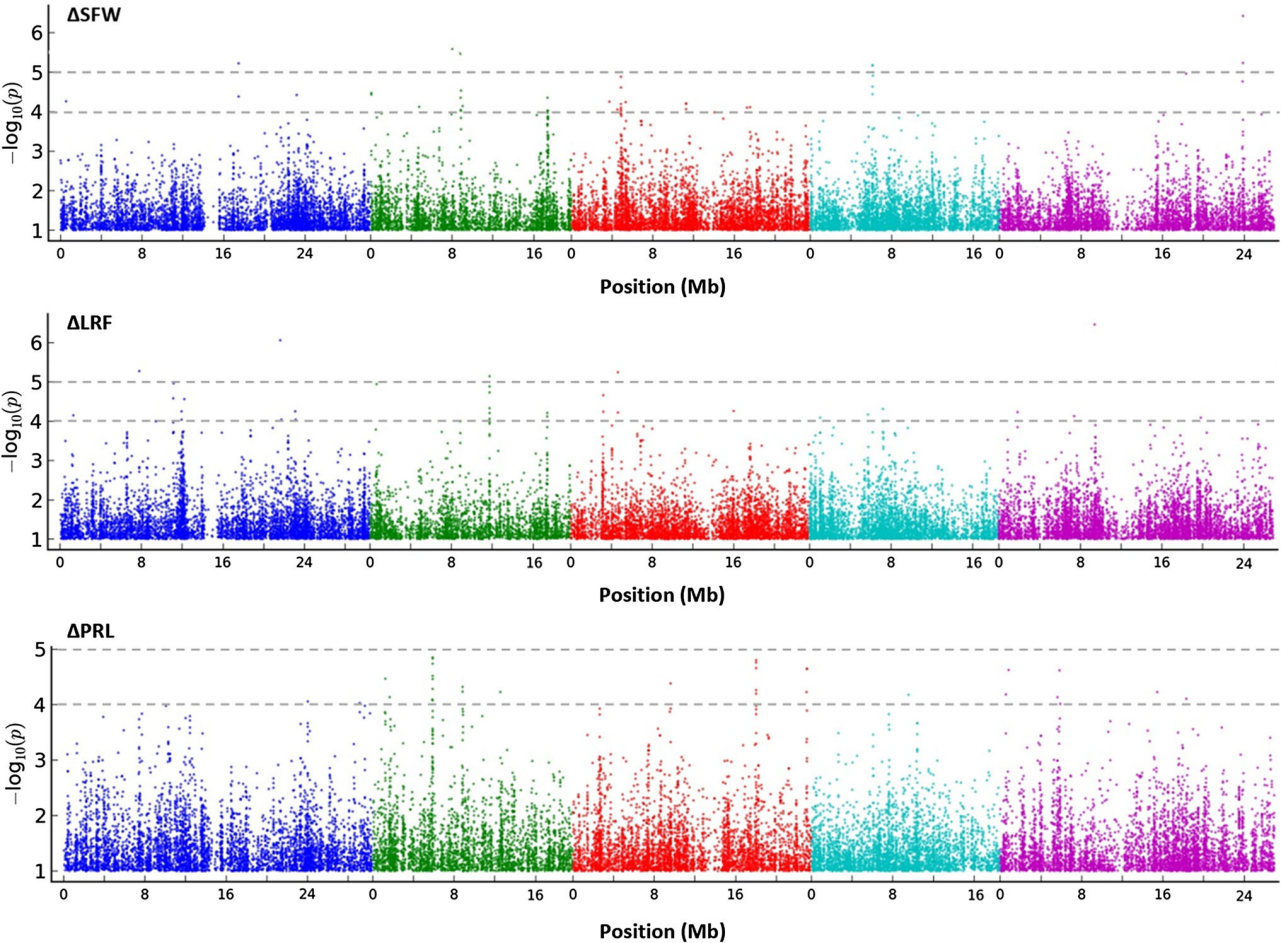
GWA mapping of *P. simiae* WCS417r-mediated effects on shoot fresh weight (∆SFW), lateral roots formation (∆LRF), and primary root length (∆PRL). Manhattan plots of the −log_10_(*P*) SNP marker–trait associations are shown. From *left* to *right*, *different colors* represent Arabidopsis chromosomes I–V. The *dotted grey lines* indicate the arbitrary thresholds of −log_10_(*P*) = 4 and −log_10_(*P*) = 5

To study the genetic basis of PGPR-mediated plant growth promotion, we performed GWA mapping on the increase in shoot fresh weight data (∆SFW), the increase in number of lateral roots formed data (∆LRF), and the change in primary root length data (∆PRL), using the 214 k SNP set that is commonly used for GWA studies in Arabidopsis (Atwell et al. 2010; Bac-Molenaar et al. 2015; Horton et al. 2012; Kim et al. 2007; Li et al. 2010). Data for ∆SFW, ∆LRF, and ∆PRL (Supplementary Table S1) were uploaded to the online tool GWAPP (http://gwas.gmi.oeaw.ac.at/) (Seren et al. 2012) to analyze the quality of the data and perform GWA mapping. The (pseudo-)heritabilities for the recorded phenotypic variation in ∆SFW, ∆LRF, ∆PRL, were 0.28, 0.62, and 0.37, respectively. After confirming that all datasets were normally distributed, they were processed with the AMM algorithm. The GWA mapping results for all three parameters are depicted in Fig. 4. Of the loci with high SNP-trait associations [−log_10_(*P*) > 5.0], 5 loci were associated with PGPR-stimulated shoot fresh weight and 5 loci with PGPR-mediated increase in lateral root formation. No loci SNPs were highly associated with PGPR-mediated changes in primary root length (Table 1; Supplemental Table S2). When using a lower threshold level [−log_10_(*P*) > 4.0], 18 additional loci were detected for PGPR-induced increase in shoot fresh weight, 20 for the PGPR-mediated increase number of lateral roots, and 20 for the PGPR-induced changes in primary root length (Supplemental Table S2). Although we observed a clear level of correlation between WCS417r-mediated changes in shoot fresh weight and the root architecture parameters (Fig. 3), none of the identified trait-associated SNPs overlapped.

**Table 1:** List of candidate genes from the GWA analysis of the *P. simiae* WCS417r-mediated effects on shoot fresh weight, lateral root formation, and primary root length of 302 natural Arabidopsis accessions

Candidate gene (AGI)	Gene annotation	Gene ontology	Growth parameter	−log_10_ *P*
At1G47603	Purine permease 19 (PUP19)	Purine transporter; may be involved in the transport of purine and derivatives such as cytokinins across the plasma membrane	∆SFW	5.22
At2G18480	Major facilitator superfamily protein	Carbohydrate transmembrane transporter activity; plant structure	∆SFW	5.58
At2G20570	Golden2-like 1 (GLK1)	Transcription factor activity; regulates expression of the photosynthetic apparatus (with GLK2)	∆SFW	5.46
At4G09730	DEAD-box protein RH39	Protein involved in chloroplast rRNA structure; involved in RUBISCO biogenesis	∆SFW	5.17
At5G59190	Subtilase family protein	Serine-type endopeptidase activity	∆SFW	6.42
At1G22080	Cysteine proteinases superfamily protein	Cysteine proteinase activity, unknown protein	∆LRF	5.28
At1G58330	ZW2, unknown protein	Similarities with RESPONSE TO ABA AND SALT 1 (RAS1)	∆LRF	6.06
At2G27450	Nitrilase-like protein 1 (NLP1)	N-carbamoylputrescine amidohydrolase; Involved in polyamine biosynthesis, nitrogen compound metabolic process	∆LRF	5.14
At3G14075	Mono-/di-acylglycerol lipase	Involved in lipid metabolic process	∆LRF	5.24
At5G26780	Serine hydroxymethyltransferase 2 (SHM2)	Serine hydroxymethyltransferase activity; involved in photorespiration	∆LRF	6.46

Shown are Arabidopsis AGI numbers of candidate genes that are located in the closest proximity of the identified highly-associated SNPs [−log_10_(*P*) > 5] for each of the measured growth parameters, their annotation and ontology in TAIR10. ∆SFW, WCS417r-induced increase in shoot fresh weight; ∆LRF, WCS417r-mediated increase in number of lateral roots formed

The linkage disequilibrium of the natural population of Arabidopsis accessions used for GWA mapping is on average 10 kb (Kim et al. 2007). Therefore, all genes within 10 kb up- or downstream of the highly [−log_10_(*P*) > 5.0] and moderately [−log_10_(*P*) > 4.0] associated SNPs were identified. Their identities, gene annotation, and functional information are provided in Supplemental Table S2. In Table 1, the highly-associated SNPs are given along with their closest candidate gene. Among the candidate genes in the closest vicinity of the high SNP-trait associations for the PGPR-mediated changes in shoot and root growth are genes encoding proteins that are associated with important plant growth processes, such as the cytokinin transporter PUP19 (Cedzich et al. 2008), the photosynthesis-related protein Golden2-like 1 (GLK1) (Waters et al. 2009), the Rubisco biogenesis-related DEAD box protein RH39 (Nishimura et al. 2010), carbohydrate transmembrane transport protein At2G18480, Nitrilase-like protein 1 (NLP1) (Kusano et al. 2008), and the photorespiration-related mitochondrial serine hydroxymethyltransferase SHM2 (Engel et al. 2011; Voll et al. 2006) (Table 1). Overall, these results provide insight in the genetic components that may contribute to the plant’s capacity to benefit from growth-promoting rhizobacteria in the rhizosphere.

## Discussion

Natural accessions of Arabidopsis have a high degree of variation in plant development, physiology, and adaptation to their biotic and abiotic environment (Alonso-Blanco et al. 2009). Using a small collection of Arabidopsis accessions, we previously explored the natural genetic variation in Arabidopsis for its capacity to develop PGPR-mediated ISR and identified quantitative trait loci related to this phenomenon (Ton et al. 1999, 2001). Haney et al. (2015) recently showed that genotypic variations in Arabidopsis accessions can influence the ability to associate with specific PGPR with consequences for plant fitness. Here we made use of the natural variation within 302 Arabidopsis accessions to investigate to what extent the different genotypes differ in their capacity to respond to growth-promoting effects of beneficial rhizosphere bacteria and to gain insight into the genetic basis of the profitable functions by which plants can benefit from their root microbiome. Analysis of the growth promotion parameters shoot fresh weight, lateral root formation, and primary root length in response to WCS417r treatment showed large natural variation among the tested accessions for all three parameters (Fig. 2). The capacity to respond to WCS417r only weakly correlated with the intrinsic growth rate of the accessions. Hence, accessions that gained more shoot fresh weight or produced more lateral roots in the mock-treatment, were not necessarily better responders to the PGPR treatment. By contrast, WCS417r-mediated changes in the growth parameters were moderately to highly correlated with each other. Especially the WCS417r-mediated increase in shoot fresh weight showed a high correlation with PGPR-induced changes in the root architecture parameters (Fig. 3), suggesting that changes in root architecture and shoot fresh weight are linked. Previously, Zamioudis et al. (2013) demonstrated that root colonization by WCS417r enhances the auxin response in the root of Arabidopsis and stimulates auxin-dependent developmental programs related to primary root, lateral root, and root hair development. Collectively, these root architectural changes enlarge the capacity of the root system to take up water and nutrients (Lopez-Bucio et al. 2003), which may contribute to the observed correlation between extra number of lateral roots formed and increased shoot fresh weight in WCS417r-treated Arabidopsis seedlings. It should, however, be noted that Arabidopsis seedlings were grown on plates in which nutrient availability was not limiting, hence we cannot rule out the possibility that the correlation between root architectural changes and increased shoot fresh weight is caused by another, so far unknown process.

In this study, we chose to treat the Arabidopsis seedlings with PGPR by co-cultivating them on the same MS agar plate while avoiding physical contact (Fig. 1). Previously, we showed that in this setup the effects on root and shoot growth are predominantly mediated by VOCs produced by the bacteria (Zamioudis et al. 2013). We realize that in this way we mainly capture the natural variation in VOCs-mediated plant growth effects in the Arabidopsis population, but because the magnitude of VOCs effects are very similar to those mediated by direct application of PGPR to the root system, we chose for this more simplified system. Also in other systems VOCs produced by PGPR have been shown to have major impacts on plant growth and development (Blom et al. 2011; Farag et al. 2013; Gutierrez-Luna et al. 2010; Park et al. 2015; Ryu et al. 2003). Microbial VOCs have been shown to stimulate auxin responses in the roots (Bailly et al. 2014; Bhattacharyya et al. 2015; Zamioudis et al. 2013), or are suggested to be taken up and utilized as e.g. sulfur nutrition (Meldau et al. 2013).

Although the PGPR-mediated increase in shoot fresh weight and changes in root architecture seem to be related in the population of natural Arabidopsis accessions tested (Fig. 3), the GWA analysis did not yield common candidate genes (Fig. 4, Table 1; Supplemental Table S2). Among the identified candidate genes for the PGPR-mediated increase in shoot fresh weight were genes associated with important plant growth processes. For instance, the *GLK1* gene encoding transcription factor Golden2-like 1 (GLK1), which together with GLK2 plays a role in the regulation of photosynthetic genes to optimize photosynthetic capacity in varying environmental and developmental conditions (Waters et al. 2009). Or the gene encoding DEAD box protein RH39, which is required for maturation of chloroplast 23S rRNA and the biogenesis of ribulose 1,5-bisphosphate carboxylase/oxygenase (Rubisco) and other chloroplast-encoded, photosynthetic proteins (Nishimura et al. 2010). Also the cytokinin transporter gene *PUP19* (Cedzich et al. 2008) is among the candidate genes associated with enhanced shoot fresh weight. Among the candidate genes related to the PGPR-mediated increase in lateral root formation is *SHM2,* which encodes the mitochondrial serine hydroxymethyltransferase SHM2 (Engel et al. 2011). The isozyme SHM1 catalyzes an essential step of the photorespiratory C2 cycle, but because *SHM2* does not complement the mutant phenotype of *shm1* and is relatively highly expressed in roots, its function is currently unknown (Voll et al. 2006). Another candidate gene related to increase in lateral root formation encodes Nitrilase-like protein 1 (NLP1). NLP1 is a N-carbamoylputrescine amidohydrolase involved in biosynthesis of polyamines that play an essential role in plant growth and survival (Kusano et al. 2008). Considering all candidate genes with SNP-trait associations in the GWA analysis, several have known or predicted functions that hold promise for being functional in mediating the plant growth-promoting effect of PGPR. Future analysis of knock-out mutants and ectopic over expressors of these genes and those with a currently unknown function should provide insight into the validity of the candidate genes and their role in the capacity of the plant to benefit from the plant growth-promoting activity of PGPR in their rhizosphere.

In summary, our survey of the natural genetic variation within Arabidopsis for the responsiveness to the plant growth-promoting activity of rhizosphere bacteria revealed that the capacity of plants to profit from their root-associated microbes has a clear genetic component. Hence, identification of genes and loci related to PGPR responsiveness in economically relevant crop species holds promise for breeding strategies that are aimed at introducing traits by which crops maximize profitable functions from their root microbiome.

## Electronic supplementary material

Below is the link to the electronic supplementary material.

Supplementary material 1 (XLSX 76 kb)

Supplementary material 2 (XLSX 68 kb)
